# Validation of a Double-Sandwich Enzyme-Linked Immunoassay for Pharmacokinetic Study of an rh-aFGF Hydrogel in Rat Skin and Serum

**DOI:** 10.3389/fphar.2020.00700

**Published:** 2020-05-19

**Authors:** Qi Hui, Rongshuai Yang, Chao Lu, Jianing Bi, Lijia Li, Jianxiang Gong, Li Zhang, Zi Jin, Xiaokun Li, Xiaojie Wang

**Affiliations:** ^1^ The Department of Dermatology of the First Affiliated Hospital of Wenzhou Medical University, Wenzhou, China; ^2^ School of Pharmacy of Wenzhou Medical University, Wenzhou, China; ^3^ Engineering Laboratory of Zhejiang Province for Pharmaceutical Development of Growth Factors, Biomedical Collaborative Innovation Center of Wenzhou, Wenzhou, China

**Keywords:** enzyme-linked immunosorbent assay (ELISA), method validation, rh-aFGF hydrogel, pharmacokinetics, relative bioavailability

## Abstract

In this study, we validated a double-sandwich enzyme-linked immunosorbent assay (ELISA) to investigate the pharmacokinetics of a recombinant human acidic fibroblast growth factor (rh-aFGF) hydrogel in rat skin and serum. A total of 130 Sprague-Dawley rats were divided into a control group, rh-aFGF hydrogel group, and a positive-control group (commercial rh-aFGF-Ai). We first determined the dilution ratio of skin homogenate and then validated the quantitative range, specificity, precision, and accuracy of our double-sandwich ELISA method, as well as the stability of our rh-aFGF hydrogel. For our pharmacokinetic study, skin and serum samples were collected at 0.5, 1, 2, 4, 6, and 10 h after rh-aFGF administration, and the concentration of rh-aFGF was measured by ELISA. The results showed that a 10-fold dilution of the skin tissue homogenate circumvented non-specific interference with endogenous proteins. The quantitative scope of the rh-aFGF calibration curve ranged from 62.5 to 4,000 pg/mL. The precision and accuracy of rh-aFGF quality-control samples were below 20%. Furthermore, bFGF, FGF21, KGF-2, and insulin did not interfere with the detection of aFGF, confirming that our method was specific. Rh-aFGF was stable under normal storage conditions. The maximum concentration (C_max_) and time to peak (T_max_) of the rh-aFGF hydrogel were 909.2 pg/cm^2^ and 0.5 h, respectively. The relative bioavailability (F) of the rh-aFGF hydrogel was 120% compared with that of rh-aFGF-Ai. The serum concentration of rh-aFGF was too low to be detected. Taken together, the pharmacokinetics of this rh-aFGF hydrogel provide further support for clinical research on rh-aFGF, and our double-sandwich ELISA method may be useful for pharmacokinetic studies of other protein-based drugs.

## Introduction

With recent advances in biotechnology, an increasing number of protein-based drugs have been applied in clinical settings and have become the first choice of treatment for many diseases (Lewis and Richard, 2015). Protein-based drugs have characteristics of high specificities, strong physiological activities, and low dosages. The processes of absorption, distribution, metabolism, and excretion of protein-based drugs *in vivo* are different from those of small-molecule drugs, which makes pharmacokinetic analysis of these drugs different from that of traditional small-molecule drugs (Luu et al., 2012; Kamath and Iyer, 2015; Shah, 2015). Especially when protein-based drugs are administered topically, their low concentrations and interference of endogenous proteins make analysis of their pharmacokinetics particularly difficult (Sun et al., 2017).

Acidic fibroblast growth factor (aFGF) is a member of the FGF protein family (Thomas, 1987), several members of which have shown promising effectiveness and safety in wound healing (Li and Li, 2007; Wu et al., 2016; Xu et al., 2016; Xu et al., 2018). Ma et al. performed a series of clinical trials that showed that topical application of rh-aFGF may represent a novel clinical treatment for deep burns and scalds (Ma et al., 2007). In addition, the emergence of many new formulations has greatly enhanced the therapeutic application of rh-aFGF. Previous research in our laboratory has shown that an rh-aFGF hydrogel significantly promotes the healing process of a full skin scald in a rat model of diabetes mellitus (Hui et al., 2018). As a macromolecular protein, rh-aFGF cannot easily diffuse through the skin barrier. However, when the skin is damaged, rh-aFGF can enter the blood circulation through the damaged epidermis. Therefore, it is necessary to study the pharmacokinetics of topical administration of rh-aFGF hydrogels to shed light on their toxicology and guarantee safety upon application.

The pharmacokinetics of protein-based drugs are generally evaluated using radioactive tracer methods (Zhou et al., 2014) and enzyme-linked immunosorbent assays (ELISAs) (Ye et al., 2015). However, these two methods often yield different pharmacokinetic results. Kuo et al. conducted pharmacokinetic analyses of human epidermal growth factor (hEGF) in rats. The half-life (t_1/2_) of unlabeled hEGF, as measured by ELISAs, was 16.1 min, while that of ^125^I-hEGF, as measured by an isotope-binding trichloroacetic-acid precipitation method, was 185 to 228 min. Furthermore, a previous study has demonstrated that radioiodination disturbs the internalization cascade of growth factors, involving receptors and cellular transduction, which in turn affects the availability of growth factors *in vivo* (Kuo et al., 1997). Therefore, in the present study, we validated a double-sandwich ELISA method and employed it to study the pharmacokinetics of topical administration of an rh-aFGF hydrogel.

## Materials and Methods

### Reagents

An rh-aFGF hydrogel, rh-aFGF stock solution, rh-bFGF, rh-FGF-21, and rh-KGF-2 were produced and provided by the Pharmacy School of Wenzhou Medical University. Commercial rh-aFGF for external use (rh-aFGF-Ai) was provided by Shanghai Tenry Pharmaceutical Company Ltd. Recombinant human insulin was provided by Liaoning Boao Biopharmaceutical Co., Ltd. RD6X buffer and Human aFGF ELISA Kit were purchased from R&D System (USA, code number was DFA00B and batch number was P155336).

### Animals and Groupings

Sprague-Dawley rats were provided by Beijing Vital River Laboratory Animal Technology Co., Ltd. The animal license number was SCXK (Beijing) 2016-0006. A total of 158 rats (190–210 g) were maintained in the animal facilities of New Drug Evaluation Co., Ltd. at the Tianjin Institute of Pharmaceutical Research (Tianjin, China), with food and water provided *ad libitum* throughout the experiment. All of the animal experiments were carried out in compliance with the guidelines issued by the Tianjin Institute of Pharmaceutical Research (Tianjin, China) and were approved by the Institutional Animal Ethics Committee.

Twenty-eight rats were used for validation of our double-sandwich ELISA method. The other 130 rats were randomly divided into the control group, rh-aFGF hydrogel group, and rh-aFGF commercial-lyophilized powder group. Rats in each group were anesthetized with 2.0% (W/V) sodium pentobarbital *via* intraperitoneal injection, after which the hair on the back of each rat was shaved. A mechanical damage model was established as follows. The rats were placed in a prone position, and a circle (2.5 cm in diameter) was drawn around the spine. Three 0.5-cm scratches were made horizontally in the marked area of each rat with a sterile surgical blade. The rats were kept in a single cage and ate and drank freely. The control group and the treatment groups were uniformly coated with 0.2 ml of normal saline, rh-aFGF hydrogel, and rh-aFGF solution on the damaged areas of rats immediately after the damage model was completed. We used the recommended clinical dosages (i.e., 100 AU/cm^2^ for the rh-aFGF hydrogel and 110 AU/cm^2^ for the commercial control rh-aFGF). Rats were euthanized under anesthesia at the indicates times during the experiment. The skin and sera of the rats were collected at 0.5, 1, 2, 4, 6, and 10 h after their treatment administrations. Skin tissue was weighed and then cut into a size of about 1 mm^3^. Tissue homogenates were prepared according to a proportion of 1 g of tissue per 5 ml of normal saline. The homogenate was centrifuged at 4°C for 10 min at 5,000 rpm. The supernatant was stored at −80°C until further use for method validation and pharmacokinetic analysis.

### Double-Sandwich ELISA

A double-sandwich ELISA was developed to measure the concentration of rh-aFGF in skin tissue homogenates and sera from rats. In brief, a 96-well microplate was pre-coated with anti-human rh-aFGF antibody (150 μL/well). An rh-aFGF quality control (QC) sample or test sample (50 μL/well) was added, and the wells were incubated at room temperature for 2 h with shaking. The liquid in the well was discarded, and wells were washed four times with washing buffer (300 μL/well). Then, an enzyme-labeled antibody (200 μL/well) was added. After incubation at room temperature for 2 h with shaking, the liquid in the well was discarded, and the washing buffer (300 μL/well) was added. The plate was washed four times, tetramethyl benzidine (TMB) substrate (200 μL/well) was added, and the plate was incubated at room temperature in the dark for 30 min. The reaction was stopped with the addition of 2 M of H_2_SO_4_ stop solution (50 μL/well). Absorbance (450 nm) was measured with a Varioskan Flash microplate reader (Thermo, USA), with a reference setting at 570 nm.

### Validation of Double-Sandwich ELISA

The double-sandwich ELISA method was validated in accordance with current recommendations for bioanalytical methods. These validation methods are described below for the skin tissue homogenate. The same processes were performed for the serum samples.

### Dilution Ratio for Skin Homogenates

In order to eliminate interference from endogenous aFGF and related proteins, we first investigated the effects of the dilution ratio of skin homogenates on the accuracy of rh-aFGF measurements. For this purpose, we used RD6X buffer to prepare a dilution gradient of skin homogenates at 1:2.5, 1:5, 1:10, and 1:20. Absorbance (450 nm) was measured with a Varioskan Flash microplate reader (Thermo, USA), with a reference setting at 570 nm.

Two different biological matrices (RD6X buffer and 10× diluted skin homogenate) were selected to prepare the calibration curve (62.5, 125, 250, 500, 1,000, 2,000, and 4,000 pg/mL). rh-aFGF QC samples and rh-aFGF test samples were diluted to three concentrations (160, 800, and 3,200 pg/mL), and the concentrations were determined with the calibration curve.

### Calibration Curve and Quantitative-Scope Analyses

A calibration curve was obtained by determining the absorbance of the rh-aFGF QC samples (62.5 to 4,000 pg/mL), and the rh-aFGF concentration in 10× diluted skin tissue homogenate was determined on the basis of this calibration curve. A four-parameter log-logistic (4-PL) model in Origin v. 7.5 software was used for fitting, with a weighting factor of 1/C2. The lower limit of quantification (LLOQ) was defined as the lowest concentration at which the precision and accuracy within 75% to 125% that could still be quantified. The upper limit of quantification (ULOQ) was defined as the highest concentration at which the precision and accuracy with 75% to 125% that could still be quantified.

### Precision and Accuracy Analyses

The within-batch precision and accuracy were assessed by measuring the concentrations of rh-aFGF QC samples (62.5, 160, 800, 3,200, and 4,000 pg/mL) on the same ELISA plate. The inter-batch precision and accuracy were assessed by measuring the concentrations of rh-aFGF QC samples on different ELISA plates using five different batches. The precision was expressed as the relative standard deviation (RSD, %), and the accuracy was expressed as the relative error (RE, %). The total error (TE, %) was defined as the sum of the within-batch and inter-batch errors.

### Specificity Analyses

FGF-21, bFGF, KGF-2, and insulin are endogenous hormones with structures that resemble that of aFGF. To detect the potential interference of these four hormones, we determined the specificity by detecting the levels of bFGF, FGF-21, KGF-2, and insulin in skin tissue homogenates from Sprague-Dawley rats of the control group. We next added rh-bFGF, rh-FGF-21, rh-KGF-2, and insulin (10 and 100 ng/mL) to the rh-aFGF QC samples (160 or 3,200 pg/mL), and determined the rh-aFGF concentration.

### Stability Analyses

Rh-aFGF QC samples (1.6 and 32 ng/mL) were added to skin tissue homogenates. The stability of rh-aFGF was determined *via* double-sandwich ELISA under the following conditions. The short-term stability of rh-aFGF was measured after storing the samples at 2°C to 8°C for 1 day or at 22°C to 26°C for 3 h. The long-term stability of rh-aFGF was measured after storing the samples at −80°C for 24 and 55 days. The protein stability after one freeze–thaw cycle was measured after storing rh-aFGF samples at −80°C and thawing at room temperature the next day. The protein stability after two freeze–thaw cycles was measured after storing rh-aFGF samples at −80°C for 15 days, thawing at room temperature, freezing at −80°C for another 15 days, and thawing again at room temperature. The rh-aFGF samples were 10× diluted in calibrator-diluent buffer on the day of measurement. Every sample was measured five times. The RE of the rh-aFGF concentration was taken as the evaluation index.

### Pharmacokinetic Analysis of rh-aFGF in Skin Tissue and Serum

After validation of the double-sandwich ELISA method, the rh-aFGF concentrations in skin tissue homogenates and serum samples were measured at the indicated time points. Pharmacokinetic parameters, namely, the time to peak (T_max_), maximum concentration (C_max_), and area under the curve (AUC_(0–t)_), were calculated *via* Phoenix WinNonlin 7.0 (Certara, USA). The relative bioavailability (F) of rh-aFGF in the skin was calculated as the ratio of the AUC_(0–10__h)_ between the rh-aFGF hydrogel and the commercial rh-aFGF-Ai.

### Statistical Analysis

Graphpad Prism v. 6.0 software was used for statistical analysis, and the obtained experimental data are expressed as the mean ± standard deviation.

## Results

### Dilution Ratios of Skin Homogenates

**Figure 1:**
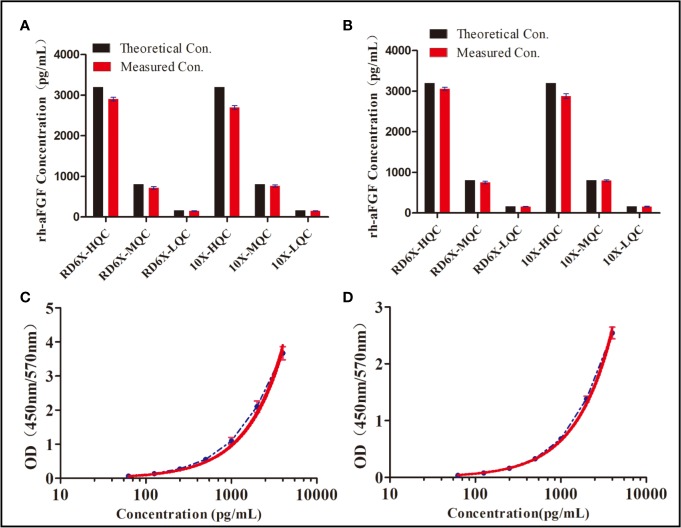
Dilution-ratio evaluation of tissue samples and establishment of a calibration curve. **(A)** Concentration of rh-aFGF quality-control (QC) samples, as calculated by the standard curve prepared with RD 6X buffer. “RD 6X” indicates the calibrator diluents of the commercial ELISA kit, which was diluted 10-fold with PBS buffer. “10×” indicates skin homogenate that was diluted 10-fold with PBS buffer. **(B)** Concentrations of rh-aFGF QC samples, as calculated by the standard curve prepared with 10× diluted skin homogenate. **(C)** Calibration curve for measurement of rh-aFGF levels in rat skin tissue homogenates (*n* = 9). The red curve is the fitted standard curve. **(D)** Calibration curve for measurement of rh-aFGF levels in rat serum samples (*n* = 8). The red curve is the fitted standard curve.

Considering that skin tissue contains endogenous aFGF and other proteins that interfere with the determination of the rh-aFGF concentration, we first studied the dilution ratio of the tissue. The absorbances of control skin homogenates diluted 2.5, 5, 10, and 20 times were 0.316, 0.179, 0.108, and 0.074, respectively. The absorbance of 10× diluted control skin tissue was approximately 0.1, which was low enough to effectively eliminate interference of endogenous proteins. To confirm the low interference of 10× diluted control tissue homogenates with the measurements of rh-aFGF levels, we prepared two calibration curves: one with RD6X buffer, and the other with 10× diluted skin tissue homogenate. As shown in **Figure 1A**, the REs of high, middle, and low concentrations of rh-aFGF samples calculated using the standard curve prepared with RD6X buffer were 93.9%, 89.5%, and 88.1%, respectively. Accordingly, the REs of high, middle, and low concentrations of rh-aFGF samples calculated using the standard curve prepared with 10× diluted skin homogenate were 93.5%, 89.8%, and 88.0%, respectively (**Figure 1B**). The accuracy was within ±20% (−17.0% to 10.2%), and the concentration ratio was in the range of 80−120% (88.0−93.9%). Therefore, interference during measurements of rh-aFGF concentrations could be neglected after skin homogenates were diluted 10-fold in RD6X buffer.

### Calibration Curve and Quantitative Scope

The calibration curve was obtained by determining the absorbance of rh-aFGF QC samples. Calibration curves of rh-aFGF levels in skin homogenates (*n* = 9) and sera (*n* = 8) are shown in **Figures 1C, D**, respectively. The fitting equation was as follows: Y = 13.667 − 13.668/(1 + (X/10382.676)^1.041^), r^2^ = 0.9999. The RE of the recalculated concentration of each rh-aFGF QC sample using the calibration curve was within the range of −1.0% to 1.5% for the skin homogenates and −1.4% to 1.7% for sera. The RSD of rh-aFGF QC samples was within the range of 1.0% to 4.0% for the skin homogenates and 1.1% to 2.6% for sera. The quantitative scope of the rh-aFGF calibration curve ranged from 62.5 pg/mL (LLOQ) to 4,000 pg/mL (ULOQ).

### Precision and Accuracy

**Figure 2:**
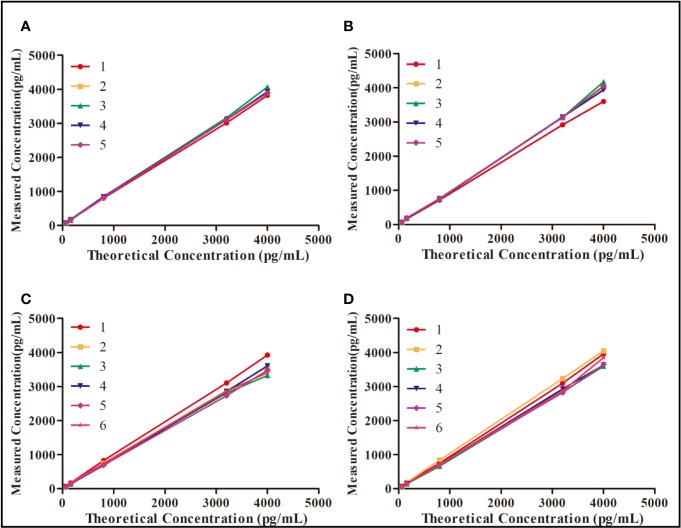
Evaluation of precision and accuracy of the double-sandwich enzyme-linked immunosorbent assay (ELISA) method. **(A)** Within-batch precision and accuracy of rh-aFGF in skin homogenate (*n* = 5). **(B)** Within-batch precision and accuracy of rh-aFGF in serum (*n* = 5). **(C)** Inter-batch precision and accuracy of rh-aFGF in skin homogenate (*n* = 6). **(D)** Inter-batch precision and accuracy of rh-aFGF in serum (*n* = 6).

The within-batch precision and accuracy of the double-sandwich ELISA method were assessed by measuring different concentrations of rh-aFGF QC samples on the same ELISA plate. Inter-batch precision and accuracy assessments were based on the measurement of rh-aFGF levels on different enzyme plates for six independent batches, and the inter-batch precision (RSD) and accuracy (RE) were calculated. As shown in **Figure 2**, the precision of rh-aFGF QC samples were within the range of 101.8% to 105.6% and 104.4% to 111.6% for with-batch and inter-batch respectively. The accuracy of rh-aFGF QC samples was within the range of 97% to 109.6% and 88.8% to 105.4% for with-batch and inter-batch, respectively.

### Specificity

**Figure 3:**
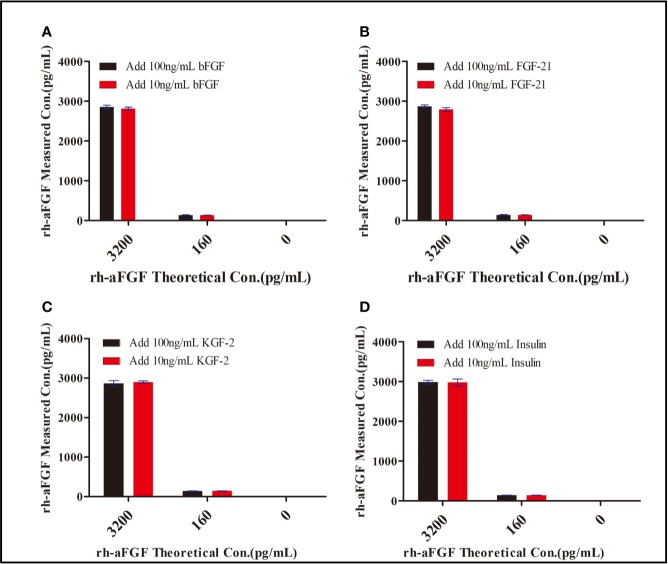
Specificity of the double-sandwich enzyme-linked immunosorbent assay (ELISA) method. **(A)** bFGF. **(B)** FGF-21. **(C)** KGF-2. **(D)** Insulin.

We chose insulin, FGF21, bFGF, and KGF-2—which show sequence homology with aFGF—to determine the specificity of the double-sandwich ELISA method. These four potentially interfering substances were not detected by our ELISA method (**Figure 3**). The RE was within the range of−17.6% to−4.3%, demonstrating that this method was specific to rh-aFGF and that the four added proteins did not interfere with the detection of rh-aFGF.

### Stability Analysis

**Figure 4:**
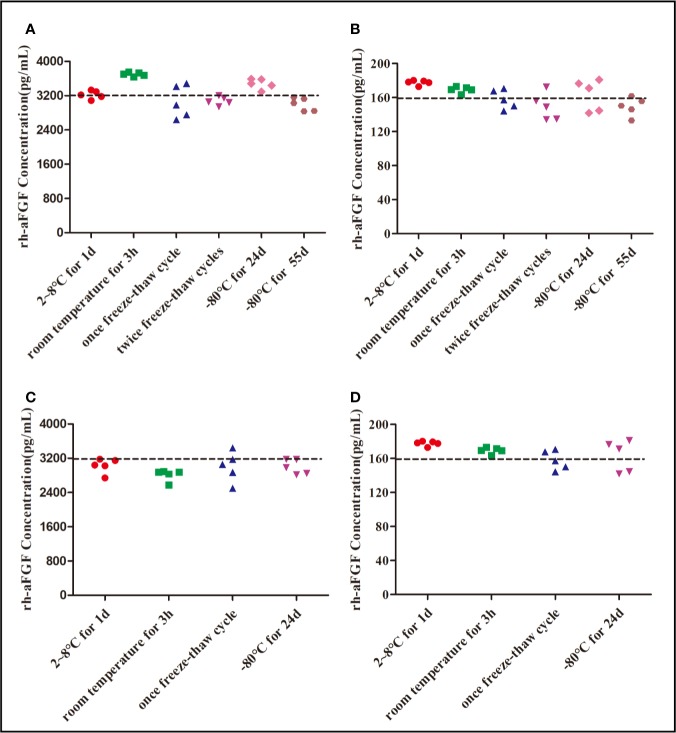
Stability of rh-aFGF samples stored under different conditions. **(A)** Stabilities of rh-aFGF HQC samples (3200 pg/mL) in skin homogenates (*n* = 5). **(B)** Stabilities of rh-aFGF LQC samples (160 pg/mL) in skin homogenates (*n* = 5). **(C)** Stabilities of rh-aFGF HQC samples (3200 pg/mL) in sera (*n* = 5). **(D)** Stabilities of rh-aFGF LQC samples (160 pg/mL) in sera (*n* = 5) from the following: (1) after 2°C to 8°C for 1 day; (2) after being at room temperature for 3 h; (3) after one freeze–thaw cycle; (4) after two freeze–thaw cycles; (5) after long-term storage at −80°C for 24 days; or (6) after long-term storage at −80°C for 55 days.

It is inevitable that rh-aFGF samples will be repeatedly frozen and thawed between collection and detection and be left at room temperature for a period of time. Therefore, it is necessary to understand the stability of rh-aFGF under different storage conditions. We studied the stability of rh-aFGF under short-term, long-term, and repeated freeze–thaw conditions (**Figure 4**). The RE of rh-aFGF QC samples was between −6.7% and 15.7% under the six following conditions: storage at 2°C to 8°C for 1 day; storage at room temperature (22−26°C) for 3 h; freeze–thawing once; freeze–thawing twice; and long-term storage at −80°C for 24 days or 55 days. Hence, rh-aFGF was considered to be stable under these storage conditions.

### Pharmacokinetic Analysis

**Figure 5:**
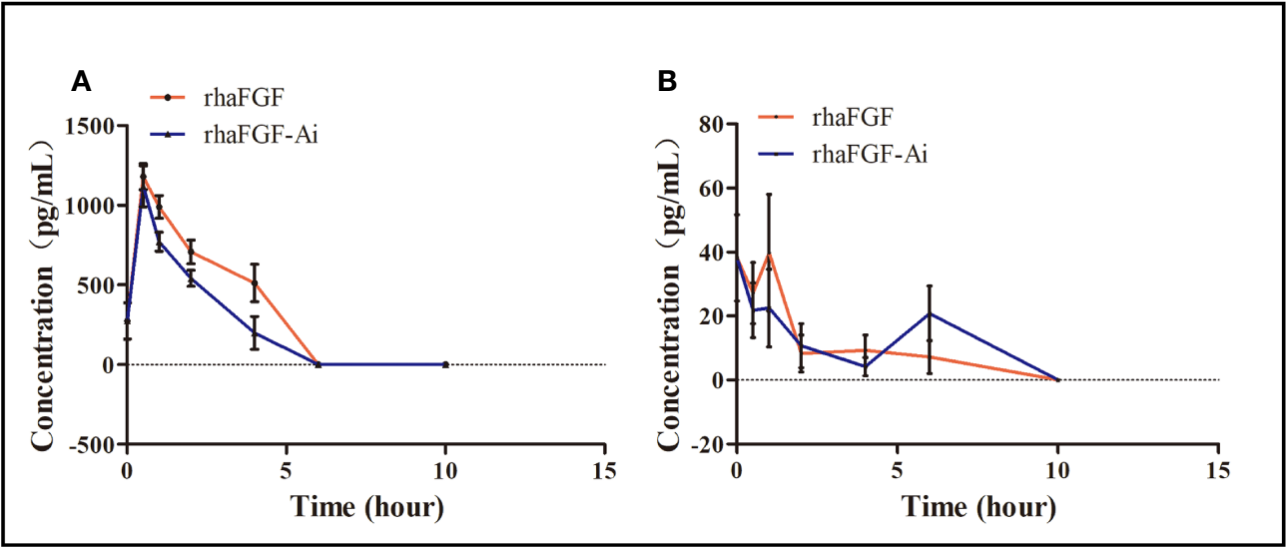
Pharmacokinetic analysis. **(A)** Drug concentration–time curve (*n* = 10) for skin homogenates. **(B)** Drug concentration–time curve (*n* = 10) for sera.

After the topical administration of rh-aFGF (100 U/cm^2^) and commercial rh-aFGF-Ai (110 U/cm^2^), the rh-aFGF concentrations in skin homogenates showed dynamic changes over time. Rh-aFGF was immediately absorbed into the skin after administration and reached maximum values at 0.5 h after administration (1179.4 ± 257.7 pg/mL for rh-aFGF and 1119.1 ± 404.2 pg/mL for rh-aFGF-Ai). After 4 h, rh-aFGF and rh-aFGF-Ai concentrations had decreased considerably (511.3 ± 372.9 and 199.3 ± 328.8 pg/mL, respectively). Rh-aFGF was not detected in the skin after 6 h (**Figure 5A**).

The average concentration of endogenous aFGF in the serum was 38.2 ± 42.7 pg/mL. After the topical administration of rh-aFGF, most animals showed little or no rh-aFGF absorption into the blood circulation (**Figure 5B**).

After topical administration, the AUC _(0–10__h)_ of rh-aFGF and rh-aFGF-Ai was 1704.1 and 1419.9 h·pg/cm^2^, respectively; the C_max_ values were 909.2 and 879.5 pg/cm^2^, respectively, and T_max_ was 0.5 h. The relative bioavailability (F), which was calculated as the ratio of AUC _(0–10__h)_ between rh-aFGF and rh-aFGF-Ai, was 120%. Therefore, after topical application of rh-aFGF and commercial rh-aFGF-Ai, the pharmacokinetic behavioral characteristics of the skin were consistent.

## Discussion

In the 1970s, ELISA was developed on the basis of immunoenzyme technology, such as immunohistochemical assays and radioimmunoassays (Zhao et al., 2012). ELISA has been widely used in medical tests and fundamental research because of its strong specificity, high sensitivity, ease of operation, and lack of radioactive contamination (Nirmala et al., 2006). Especially in the study of pharmacokinetics of recombinant protein-based drugs, ELISA has become an important method for measuring the concentrations of target proteins in sera or other tissue samples. Although ELISA exhibits the above advantages, drawbacks include many endogenous interference factors and its low automation compared with that of HPLC and other analytic methods. Therefore, when using ELISA, it is necessary to optimize and validate the test conditions according to the *ad hoc* purpose of the experiment to ensure that its application satisfies all requirements (Puszkiel et al., 2017). When using ELISA to study pharmacokinetics of a recombinant protein, the interference of serum or other biological substrates must be analyzed.

To ensure that our double-sandwich ELISA method met the requirements for pharmacokinetic studies of rh-aFGF, we validated the quantitative range, accuracy, precision, and specificity of our method, as well as the stability of the employed protein (Cespedes et al., 2006; Zhang et al., 2014; Buono et al., 2019). In addition, because rat serum and skin tissue samples contain endogenous growth factors that can interfere with the detection of rh-aFGF, we first validated the optimal dilution ratio of skin tissue homogenates. In this step, rh-aFGF levels in QC samples at high, medium, and low concentrations were calculated by calibration curves that were made using dilutions in RD6X buffer or in 10× diluted skin tissue homogenate. The ratios obtained for the three QC samples were 93.9%, 89.5%, and 88.1%, which conform to the technical requirements of the guiding principles for the validation of quantitative analysis methods for biological samples. Therefore, our employed ELISA method excluded any influence from endogenous substances on rh-aFGF detection and yielded high accuracy. Considering that FGF protein family members have some sequence homology amongst one another, we added low and high concentrations of bFGF, KGF-2 (FGF10), FGF21, and insulin to the tissue homogenates to analyze the specificity of our method. The results showed that bFGF, FGF-21, KGF-2, and insulin did not interfere with the measurement of aFGF concentrations.

The standard curve for concentration measurements is usually drawn using linear regression with a four-parametric equation. When the abscissa is log-transformed, the standard curve is S-shaped, having an upper and lower platform. Therefore, the concentration range of the standard curve should be in the middle section of the S curve. If the quantitative range is too wide, accuracy cannot be achieved. In the same way, if the quantitative range is too narrow, repeated dilution is needed to make the sample concentration fall within the range of the standard curve. Therefore, it is necessary to determine the scope of the standard curve at the beginning of the establishment of the method. In our present study, the quantitative scope of the rh-aFGF calibration curve was between 62.5 pg/mL (LLOQ) and 4,000 pg/mL (ULOQ), which was in the middle of the calibration curve. The accuracies (RE) of the ULOQ and LLOQ concentrations were within the range of −12.6% to 2.3% and −13.8% to 22.2%, respectively, which also conformed to the standard, indicating that our established approach has good selectivity for the determination of rh-aFGF levels. Thus, through comprehensive validation, the sensitive and accurate ELISA method established in the present study may facilitate pharmacokinetic analysis of the topical administration of rh-aFGF. In our present pharmacokinetic analysis, we found high variation in drug concentrations over time, with an early peak (0.5 h) and no drug detected at 6 to 10 h after administration. Moreover, we observed no evident increase in the serum rh-aFGF concentration, demonstrating irregular dynamic volatility over time. These results confirm that the pharmacokinetic behavioral characteristics of the hydrogel formulation were in good accordance with those of the commercial rh-aFGF. Moreover, the relative bioavailability of the rh-aFGF hydrogel formulation was 120% compared with that of commercial rh-aFGF. Commercial rh-aFGF lyophilized powder has been used in the clinic for a long time and its safety has been proven. Therefore, comparison of the bioavailability between our rh-aFGF hydrogel with commercial rh-aFGF-Ai may help us to better determine the safety of our rh-aFGF hydrogel. In a previous study, a rh-aFGF hydrogel showed stronger wound-healing-promoting effects in a rat model of type-2 diabetes, which may be related to its higher bioavailability (Hui et al., 2018).

In summary, we validated a double-sandwich ELISA method to quantify the concentration of rh-aFGF in rat skin tissue homogenates. Our validated double-sandwich ELISA method was then used to determine the pharmacokinetics of our rh-aFGF hydrogel upon topical application, providing further support for future research on its safety and toxicology. Moreover, our validated double-sandwich ELISA method may also be useful for pharmacokinetic studies of other protein-based drugs.

## Data Availability Statement

The datasets generated for this study are available on request to the corresponding authors.

## Ethics Statement

The animal study was reviewed and approved by Tianjin Institute of Pharmaceutical Research. All animal experiments were carried out in compliance with the guidelines issued by the Tianjin Institute of Pharmaceutical Research (Tianjin, China) and were approved by the Institutional Animal Ethics Committee.

## Author Contributions

QH and RY carried out the studies and collected data. CL, LZ, and LL performed the statistical analysis and participated in its design. JB, JG, and ZJ participated in acquisition and analysis of data. XL and XW designed the study and wrote the manuscript. All of the authors read and approved the final manuscript.

## Funding

The present study was supported by the National Natural Science Foundation of China (No. 81601695); Science and Technology Project of Wenzhou (No. Y20180150).

## Conflict of Interest

The authors declare that the research was conducted in the absence of any commercial or financial relationships that could be construed as a potential conflict of interest.

## Abbreviations

aFGF, acidic fibroblast growth factor; AUC, area under the curve; Cmax, maximum concentration; ELISA, enzyme-linked immunosorbent assay; hEGF, human epidermal growth factor; HQC, high-quality Control (3,200 pg/mL); LLOQ, lower limit of quantification; LQC, low-quality control (160 pg/mL); QC, quality control; RE, relative error; RSD, relative standard deviation; t1/2, half-life; TE, total error; Tmax, time to peak; ULOQ, upper limit of quantification.
